# Strain US Elastography for the Characterization of Thyroid Nodules: Advantages and Limitation

**DOI:** 10.1155/2015/908575

**Published:** 2015-04-14

**Authors:** Vito Cantisani, Hektor Grazhdani, Elena Drakonaki, Vito D'Andrea, Mattia Di Segni, Erton Kaleshi, Fabrizio Calliada, Carlo Catalano, Adriano Redler, Luca Brunese, Francesco Maria Drudi, Angela Fumarola, Giovanni Carbotta, Fabrizio Frattaroli, Nicola Di Leo, Mauro Ciccariello, Marcello Caratozzolo, Ferdinando D'Ambrosio

**Affiliations:** ^1^Department of Radiology, Anatomopathology and Oncology, Sapienza University of Rome, Viale del Policlinico 155, 00161 Rome, Italy; ^2^Venizelio Regional General Hospital of Heraklion, Leoforos Knosou, 714 09 Iraklio, Greece; ^3^Department of Surgical Sciences, Sapienza University of Rome, Viale Regina Elena 324, 00161 Rome, Italy; ^4^University of Tirana, Rruga Arben Broci, Tirana, Albania; ^5^University Hospital of Pavia, Viale Camillo Golgi 19, 27100 Pavia, Italy; ^6^Department of Medicine and Health Sciences, University of Molise, Contrada Tappino, 86100 Campobasso, Italy; ^7^Department of Experimental Medicine, Sapienza University of Rome, Viale Regina Elena 324, 00161 Rome, Italy

## Abstract

Thyroid nodules, with their high prevalence in the general population, represent a diagnostic challenge for clinicians. Ultrasound (US), although absolutely reliable in detecting thyroid nodules, is still not accurate enough to differentiate them into benign and malignant. A promising novel modality, US elastography, has been introduced in order to further increase US accuracy. The purpose of this review article is to assess the thyroid application of US strain elastography, also known as real-time elastography or quasistatic elastography. We provide a presentation of the technique, and of up-to-date literature, analyzing the most prominent results reported for thyroid nodules differentiation. The practical advantages and limitations of strain elastography are extensively discussed herein.

## 1. Introduction

Thyroid nodules are reported to be found in 33% of unselected adults between the age of 18 and 65 years and in 50% of the population of over 65 years of age [1, 2]. Although the majority of the thyroid nodules are benign, malignancy has a prevalence of 5%–15% [3]. Ultrasound (US) is accurate in the detection of thyroid nodules, but it has a relatively low diagnostic performance for the differentiation between benign and malignant nodules [4–6]. US sensitivity and specificity in characterizing thyroid nodules vary considerably from study to study and range between 52 and 97% and 26.6 and 83%, respectively [7, 8]. According to the American Thyroid Association guidelines, no single US feature or combination of features is adequately sensitive or specific to identify all malignant nodules [9]. For this reason, fine needle aspiration biopsy (FNAB) is required for the nodules greater than 10 mm or those with suspicious ultrasound signs [9–12]. However, FNAB has inherent limitations, with specificity of 60–98% and a sensitivity ranging from 54% to 90% in various studies [13–16] due to indeterminate and nondiagnostic results. As a consequence, a significant number of patients eventually receive unnecessary thyroid surgery. Therefore, improvement and refinement of noninvasive methods to depict malignancy are needed.

In this context, US elastography (USE) has recently been introduced in the clinical workup of thyroid nodules.

USE is a US-based technique to assess the biomechanical properties of tissue in the clinical setting. Among different types of USE, strain USE was the first to be introduced into commercially available systems. It is based upon the principle that, under compression, the softer parts of tissues deform easier than the harder parts [17]. The concept of USE was firstly conceived and realized in 1991 by Ophir et al. [18] and gradually developed into a robust US examination method. It has recently gained great interest and attention and has found rapid diffusion in various diagnostic applications, including the thyroid nodules [19, 20]. As shown by a number of studies, USE of thyroid nodules seems promising in differentiating benign from malignant nodules [21–25]. The American Thyroid Association guidelines in 2009 stated that USE is an emerging and promising technique that requires additional validation with prospective studies [9].

The aim of this review paper is to present the role of strain USE in the differential diagnosis of thyroid nodules based on the available evidence retrieved by systematic literature search of the MEDLINE, EMBASE, and COCHRANE databases including the guidelines established by international societies.

## 2. Physical Principles and Technique of Strain USE

A deformation force is applied to tissue resulting in changes in dimensions and shape, which are then used to calculate the stiffness of the tissue. This is the underlying physical mechanism on which all forms of current commercially available USE methods are based. However, the alternative technologies differ according to the method used to deform tissue and the way they display deformation, leading to 3 main types of USE: strain USE, acoustic radiation force impulse (ARFI), and shear wave USE. Detailed information on the physics and technology of the different types of USE is available in part 1 of the EFSUMB guidelines and recommendations for the clinical use of elastography [26].

Strain USE detects the local deformation (strain) under slight pressure and displays it as a relative value in comparison to the strain values of the different tissues within the region of interest. Strain USE is also named real-time ultrasound elastography (RTE), or strain elastography (SE), or free-hand elastography and is the most widely available type of USE. The pressure is performed either by the hand held US transducer or by physiological movements (e.g., carotid pulsation). This results in the elastographic image, also known as elastogram, which is represented as a color coded image superimposed on the B-mode image and displayed next to it on the screen. The quality of the operator's free-hand pressure is visualized on the screen as a sine-wave or displayed with a numerical scale, allowing the operator to assess the validity of the compression cycles in real-time. For computing strain images without noise, the light and cyclic probe pressure has to be harmonic with a near constant rate of displacement [26]. In general, a rectangular, or elliptic, or rounded region of interest (ROI) is used, large enough to include the entire nodule as well as a large portion of the surrounding thyroid and perithyroid tissue. This technique allows a qualitative and a semiquantitative assessment of nodule elasticity. The qualitative assessment (elastogram) represents a mapping of the amount of tissue strain at each location [18, 27]. Color coding depends on the system and usually blue represents hard, stiff tissue (with lowest elastic strain or no strain), red represents soft tissue (with greatest elastic strain), and green or orange represents intermediate level of stiffness. There is also a semiquantitative measurement method (the strain ratio), which represents the ratio of strains of the area of interest (ROI) to an equally measuring area in the reference tissue.

## 3. Qualitative USE Scoring Systems of Thyroid Nodules

**Figure 1:**
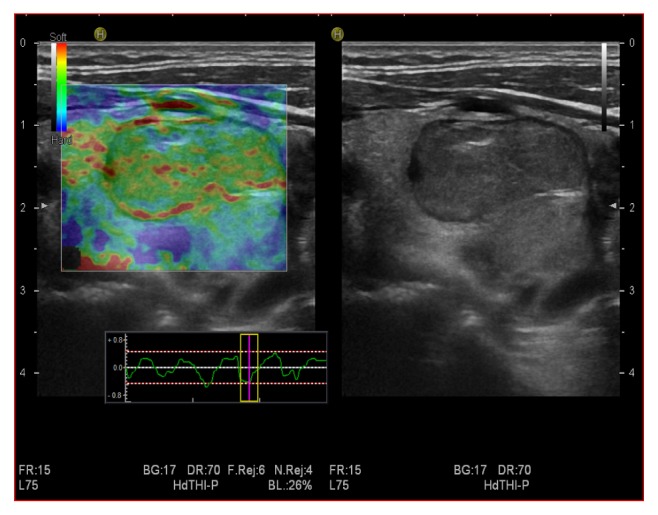
Benign thyroid nodule that appeared soft at SE, with score 2.

**Figure 2:**
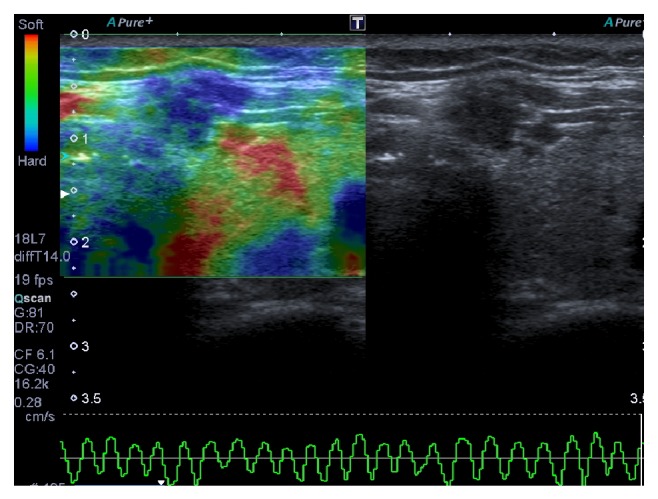
Malignant thyroid nodule that appeared hard at SE, with score 4.

**Figure 3:**
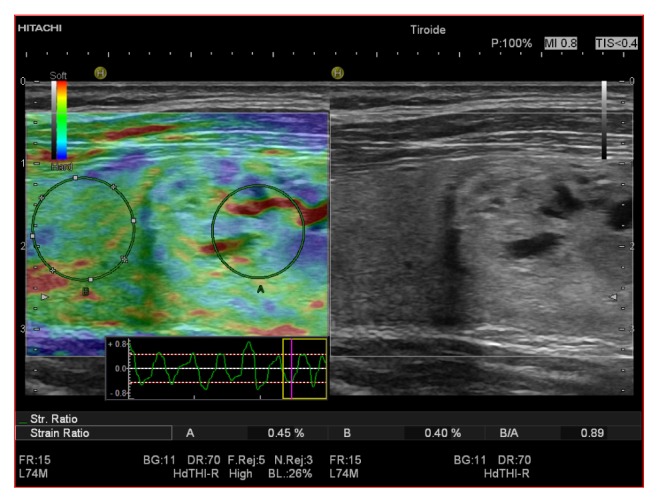
Benign thyroid nodule that appeared soft at SE, with a strain ratio of 0.89 which corresponded with final histological diagnosis of benign nodule.

Strain elastograms of nodules are qualitatively evaluated with a stepwise scoring system, according to the prevalent color in the nodule. The two principal scoring systems are those classified by Asteria et al. [28] and Rago et al. [29]. The first one, based on the breast strain USE scale of Itoh et al. [30], includes four different patterns [28] (Figure 3). The thyroid nodules with scores 1 and 2 are considered benign (Figure 1) and those with scores 3 and 4 are classified as suspicious for malignancy [28] (Figure 2). However, some authors have found that assigning benignity to score 3 further increases the specificity of the method for cancer detection [31].

The strain USE scale of Rago et al., based on the breast strain USE scale of Ueno and Itoh [27], classifies nodules on a scale of 1 to 5. The first three scores are considered as suggestive of benignity and scores of 4 and 5 are classified as suspicious for malignancy [29].

In cases of a complex cystic-solid lesion, only the solid component of the nodule is examined [31], taking care to exclude artifacts caused by the cyst [26].

A modified Asteria scale was used by Rubaltelli et al. both for thyroid nodules [32] and neck lymph nodes [33]. It consists of a five-step system that divides Asteria score 3 into patterns 3A and 3B, with a scale description as follows. Pattern 1: the entire nodule section is diffusely elastic. Pattern 2: the formation appears to be largely elastic with the inconstant appearance of anelastic areas during the real-time examination. Pattern 3: constant presence of large anelastic areas is seen at the periphery (Pattern 3A) or center (Pattern 3B) of the formation. Pattern 4: uniformly displayed anelasticity throughout the whole nodule. Lesions that present Pattern 1 or 2 are classified as probably benign, while Patterns 3 and 4 are indicative of probable malignancy [32, 33].

## 4. Semiquantitative Approach: Strain Ratio (SR)

**Figure 4:**
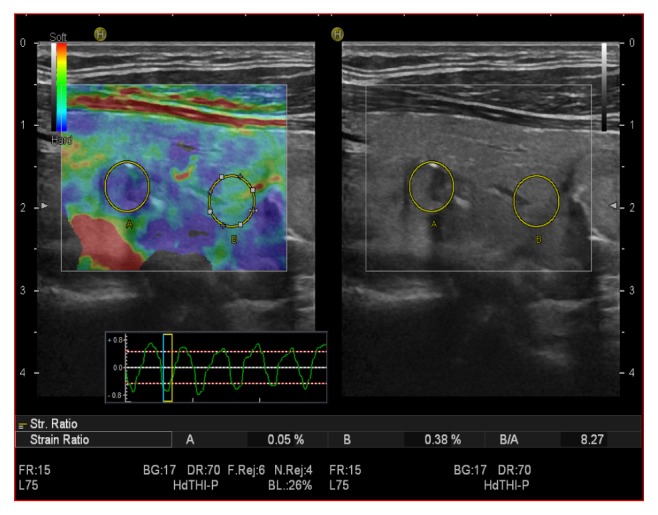
Malignant thyroid nodule that appeared hard at SE, with a strain ratio of 8.27, in the malignant range.

In an attempt to achieve a standardized and objective stiffness evaluation, a numeric parameter, the strain ratio, was introduced [34, 35]. The strain ratio is a semiquantitative analysis that compares the stiffness or strain of two different areas within the same image: two regions of interest (ROIs) are manually applied on the screen, one on the target lesion and the second on the reference normal thyroid, allowing the calculation of their strain ratio by the immediate real-time US-machine analysis [36] (Figures 3 and 4).

## 5. Carotid Pulsations Thyroid Elastography

Carotid artery pulsation can be used as the compression source for thyroid SE. This method was first introduced by Bae et al. in a preliminary study indicating the feasibility of the pulsation-induced thyroid SE [37] and was later assessed by various studies [36–42]. This method has the advantage that no free-hand external compression is used and therefore is less operator-dependent. It is both qualitative (color coded elastograms) and semiquantitative with strain ratio computations. The operator holds the probe over the thyroid applying no pressure and the anteroposterior displacement of thyroid tissue caused by the carotid pulsation is followed with a motion tracking technique and used to calculated the strain images (elastograms) [36, 42].

## 6. Diagnostic Performance of USE

For the differentiation of malignant and benign thyroid nodules, a number of literature reports show encouraging results for SE. The diagnostic performances of the main studies are presented in Table 1.

In 2010 a meta-analysis of 8 studies including a total of 639 thyroid nodules resulted in encouraging results. An overall mean sensitivity of 92% (confidence interval 88–96%) and mean specificity of 90% (confidence interval 85–95%) were shown with a significant heterogeneity found for specificity in the different studies [43].

However, the first encouraging results were challenged by a large retrospective study of Moon et al. [44] with 703 nodules (217 malignant). SE was assessed with both Asteria and Rago scoring criteria, but the results showed inferior performance of elastography (sensitivity 65.4% and negative predictive value (NPV) 79.1%), compared with gray-scale US features in combination (sensitivity 91.7% and NPV 94.7%), so the authors concluded that SE was not useful in recommending FNAB. Similarly, discouraging results are reported in another study of 2012 [45] with 237 thyroid nodules (58 malignant) that reported lower performance of RTE in comparison with gray-scale US.

On the other hand, in 2013, however, the study group of Azizi et al. [31] in a prospective study using four-grade elasticity score in the evaluation of 912 nodules resulted in positive predictive value (PPV) of 36.1%, which was slightly higher than that of microcalcifications (35.9%) and significantly greater compared with hypoechogenicity (13.6%) and isthmus location (16.9%). The negative predictive value (NPV) of elasticity score was 97.2%, which was better than any other predictor for malignancy. This study involved the greatest number of nodules ever to be systematically studied with SE and furthermore it had no patient selection bias unlike the two above mentioned studies.

Another meta-analysis published in 2013 [46] included 24 studies with 2624 patients and 3531 thyroid nodules (927 malignant and 2604 benign). Their statistical analysis yielded diagnostic performance measures which were better for SE than for US features. The sensitivities and specificities were, respectively, as follows: elasticity score, 82% and 82%; strain ratio, 89% and 82%; hypoechogenicity, 78% and 55%; microcalcifications, 50% and 80%; irregular margins, 66% and 81%; absent halo sign, 56% and 57%; nodule vertical development, 46% and 77%; and intranodular vascularization, 40% and 61%. They concluded that USE increased US accuracy. The differences in the results of the above studies are likely to result from differences in the frequency of malignancy within the study groups as well as the specific technology employed. The risk of malignancy in the study by Moon et al. was 30% [44], while in the one by Azizi et al. it was 11.40%, indicating that very different populations were being evaluated [31]. Furthermore, Moon et al. excluded complex lesions with >20% cystic component or lesions containing macrocalcifications [44].

Another issue to be addressed is whether a US and SE combined evaluation improves the diagnostic performance for thyroid cancer detection. According to Moon et al. [44] SE does not improve diagnostic performance. However, in another study by Trimboli et al. [47], based on a prospective evaluation of 498 thyroid nodules, the combination of US features with the SE four-class color scale yielded improvement with 97% sensitivity and 97% NPV whereas US alone had 85% sensitivity and 91% negative predictive value. The authors suggest that, by adding SE evaluation, the sensitivity for malignancy of US findings is markedly increased and the selection of nodules that do not need cytology is made more reliable. SE has been found promising even in small solid thyroid nodules (<10 mm in maximum diameter) that give indeterminate results on conventional ultrasound [48].

Another issue is the role of SE in the evaluation of thyroid nodules of indeterminate FNAC [49–51]. In two studies [47, 48] SE was found useful for the characterization of nodules with indeterminate or nondiagnostic cytology and therefore potentially useful in selecting patients who are candidates for surgery. However, these results were not confirmed in another study which suggested the need for quantitative analytical assessment of nodule stiffness to improve USE efficacy [49].

Regarding the reproducibility of SE for thyroid nodules, Park et al. found less interobserver agreement for SE compared with gray-scale US in the diagnosis of malignant thyroid nodules [22], whereas Kim et al. found substantial intra- and interobserver agreement for SE [52].

## 7. Diagnostic Performance of Strain Ratio (SR)

Several studies have evaluated the accuracy and role of SR for the detection of malignant thyroid nodules (Table 2).

A recent study [53] involving 344 thyroid nodules evaluated the accuracy and interoperator agreement of SR for the detection of malignancy. The authors found a sensitivity of 93% and a specificity of 92% for the expert operator and excellent interoperator agreement. Data were unaffected by nodule size or thyroiditis. These findings were confirmed in multivariate analysis demonstrating a significant correlation of the SR with malignancy.

Chong et al. [54] found that although US elastography is helpful to predict malignant thyroid nodules, the addition of SR to color mapping does not improve performance compared to color mapping alone. They also found that a ratio higher than 1.21 may serve as the best cut-off value for predicting malignancy. However, according to the meta-analysis published in 2013 [46], SR performed slightly better than qualitative USE and any other US features.

Another issue is the effect of different technical parameters in the evaluation of SR. Havre et al. [24] performed a study on a US phantom and found that the SR depends only on the position of the ROI to the reference area and not on the size and the dynamic range, thus providing better reproducibility in comparison with the qualitative scoring which is influenced by the preselected elasticity dynamic range. Therefore care should be taken to always position the reference ROI in normal parenchyma in the same depth as the examined nodule, even though the ROI may be very small due to limited normal thyroid. In cases of multinodular thyroid with no normal parenchyma the reference ROI can be positioned in the contralateral lobe but always in the same depth.

The SE method, employing the carotid artery pulsation combined with a semiquantitative evaluation of stiffness, seems to provide promising results [21, 36, 38–40]. Lim et al. using this technique found that thyroid elastography with intrinsic compression can produce reliable results compared with external compression elastography [36]. Various other metrics have been proposed using carotid artery pulsation SE with encouraging results. Using the standard deviation of strain within a thyroid nodule during diastole (diastolic strain variation index (DSVI)) or systole (systolic thyroid stiffness index (STSI)), a reduction in the number of FNAs by 53% and 60.8%, respectively, can be achieved [39, 40]. A semiquantitative SE contrast index was effective in distinguishing small papillary thyroid carcinomas [41].

## 8. Limitations

The limitations of SE are a result of technical issues associated with the application and physics of the technique as well as the histological features of the nodules, leading to misinterpretations and pitfalls.

SE in all its forms remains an examiner-dependent method. All SE techniques require a trained and experienced operator to perform valid free-hand cyclic compressions that can yield reliable and reproducible SE readings. The free-hand probe pressure is difficult to standardize among different US operators and strain variations due to changes in the amplitude and velocity of compression that cannot be avoided. Nonuniform compressions produce intra- and interobserver variability [22]. Therefore, several compression-relaxation cycles are needed to ensure that quality data are obtained [22, 43, 49, 50]. Another important issue is the fact that prestress compression can result in misleadingly high stiffness results, especially in superficial tissues like the thyroid [24, 55]. Therefore, the operator should be trained to maintain just a light contact and pressure before beginning the cycles of palpations because tissues appear stiffer when they are precompressed [24]. Another technical limitation is the lack of standardization both in the technique application, the type of measurements obtained, the cut-off values, and the color coding [17, 47].

The existence of carotid artery pulsations generates variable tissue deformations and the ratio index may be different depending on the presence of arrhythmia, atherosclerotic changes, and hypertension [43, 53], leading to potential pitfalls in the SE evaluation [17, 47]. Another limitation is that strain values in a lesion may cover a larger range than that displayed on the color coded elastogram [17]. For this reason, an elastogram optimized for a rather soft material will not accurately display the variations in hard materials, leading to a need for special presets for thyroid SE.

Besides inherent technical limitations, the histological features of the nodules themselves may lead to pitfalls. Fibrosis within a nodule can be a confounding factor in elasticity imaging. There is no study evaluating fibrosis in thyroid nodules using SE; however, many studies on liver SE have proved a correlation between levels of stiffness and fibrosis [56]. Fibrosis may be a feature in both benign and malignant nodules and could therefore be a misleading factor. The presence of autoimmune thyroiditis seems not to influence the SE results [53], whereas calcifications, partially cystic or colloid components, isthmus location, nodule size, and the presence of multinodular goiter are correlated to increased levels of stiffness [25, 53, 57]. Follicular carcinomas may lead to false negative results in SE, as they may be soft and therefore may be missed with SE [43].

## 9. Future Prospects

**Figure 5:**
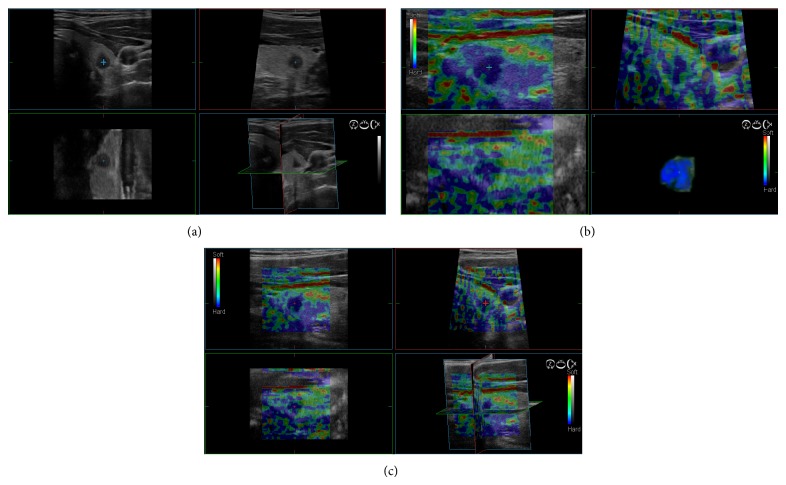
An example of a malignant thyroid nodule at 3D US and 3D SE; (a) the lesion appeared hypoechoic with ill marginated margins on three-axis planes; (b) and (c) the lesion appeared completely anelastic corresponding to score 4 at 3D USE with better delination of irregularity of the margins.

**Figure 6:**
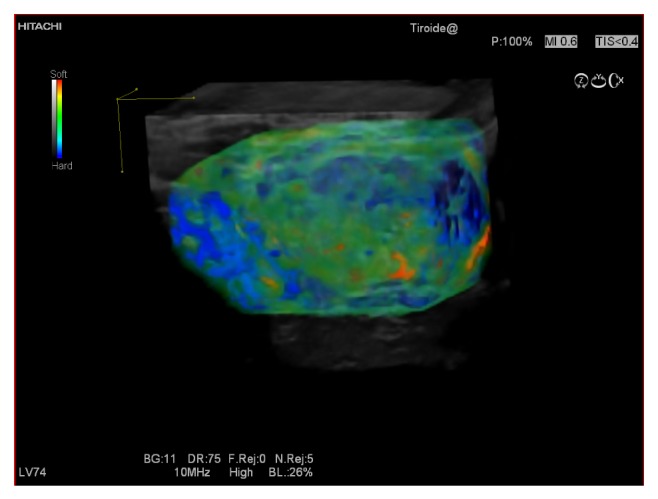
An example of a benign thyroid nodule which at 3D USE showed score 2.

SE is expected to technically evolve in the following years. Volumetric 3D elasticity images with 3D probes are currently being developed resulting in high-resolution 3D strain-volumetric images [58]. Initial data in thyroid as well as breast and testis show that in vivo 3D strain imaging is feasible and may have the potential to reduce noise and help to differentiate cystic and solid lesions [59] (Figures 5 and 6). More work on standardization of the technique and dedicated thyroid SE protocols are expected to develop in the future in order to overcome the limitations associated with the above factors.

## 10. Conclusions

According to the EFSUMB guidelines on SE, the technique may be considered, in expert hands, a useful complement to US, enhancing its accuracy for thyroid malignancy detection. EFSUMB guidelines also reported that elastography may be used to guide the followup of lesions negative for malignancy at FNA. Given the high prevalence of thyroid nodules and the substantial costs related to their workup and management, the use of SE could be a valuable tool for a better selection of nodules that need FNAB. Large multicenter studies and periodic evaluation by international experts' consensus panels are necessary to establish the role of SE in the diagnostic workup of thyroid nodules.

## Figures and Tables

**Table 1:** Diagnostic performance in malignancy detection, of SE with color coded scale for elasticity evaluation, in selected studies.

Study	Number of nodules	Sensitivity %	Specificity %	Reference standard
Rago et al., 2007 [29]	92	97	100	Surgery
Asteria et al., 2008 [28]	86	94	81	FNAB or surgery
Tranquart et al., 2008 [60]	108	100	93	FNAB
Hong et al., 2009 [23]	145	88	90	Surgery
Rubaltelli et al., 2009 [32]	51	82	86	FNAB or surgery
Lippolis et al., 2011 [49]	102	89	6	Presurgery of indeterminate cytology (follicular)
Moon et al., 2012 [44]	703	65	58	FNAB or surgery
Azizi et al., 2013 [31]	912	80	70	FNAB or surgery
Ko et al., 2014 [61]	367	89	81	FNAB or surgery
Mehrotra et al., 2013 [62]	146	90	79	FNAB or surgery

**Table 2:** Diagnostic performance of semiquantitative SE with strain ratio for elasticity evaluation, in selected studies.

Study	Number of nodules	Sensitivity %	Specificity %	Reference standard
Dighe et al., 2008 [38]^∗^	62	100	79	FNAB or surgery
Vorländer et al., 2010 [63]	309	43.2	Not referred	Surgery
Cakir et al., 2011 [64]	391	73	70	Surgery
Cantisani et al., 2014 [53]	354	93	92	FNAB or surgery

^∗^Dighe et al. [38] used carotid artery compression SE.

## Abbreviations

AUC: Area under the ROC curve
ECI: Elasticity contrast index
FNAB: Fine needle aspiration biopsy
NPV: Negative predictive value
PPV: Positive predictive value
ROI: Region of interest
ROC: Receiver operation characteristics
RTE: Real-time elastography
SE: Strain elastography
SR: Strain ratio
US: Ultrasound
USE: Ultrasound elastography.
